# Biogenesis, characterization, and applications of *Spirulina* selenium nanoparticles

**DOI:** 10.1186/s12934-025-02656-6

**Published:** 2025-02-07

**Authors:** Asmaa S. Yassein, Rokaia B. Elamary, Eman A. Alwaleed

**Affiliations:** 1 Faculty of Science, Botany and Microbiology Department, South Valley University, Qena, 83523 Egypt; 2 Faculty of Science, Botany and Microbiology Department, Luxor University, Luxor, Egypt

**Keywords:** SP-SeNPs, Antioxidant, Antimicrobial, Anticoagulant, Cell viability, Anti-inflammatory

## Abstract

**Background:**

Nowadays, researchers are attracted to the phyco-synthesis of selenium nanoparticles (SeNPs) for biotechnological and medical applications as they possess many advantages such as safety, nutritional value, and easy biodegradation than gold, copper, and silver nanoparticles. *Spirulina platensis* is the preferred microalgae for SeNPs synthesis because it contains many compounds that increase their stability making them fit for biomedical treatments.

**Results:**

The biosynthesized *Spirulina platensis* selenium nanoparticles (SP-SeNPs) were spherical and crystalline, with a diameter of 65 nm and a net charge of -16.7 mV. Furthermore, they were surrounded by active groups responsible for stability. The DPPH radical scavenging test assessed the antioxidant efficacy of SP-SeNPs and exposed scavenging inhibition of 79.234% at a 100 µM dosage. ABTS and H_2_O_2_ radical scavenging assay is dose-dependent recording IC50 of 50.69 and 116.18 µg/ml, respectively. The antibacterial efficacy was investigated against 13 G-negative & G-positive bacteria. The study demonstrated that SP-SeNPs had antibacterial and antibiofilm efficiencies against the tested strains with MBC of 286–333 µg/ml. The highest percentages of biofilm inhibition were recorded for *Bacillus subtilis* and *Klebsiella pneumoniae,* with ratios of 78.8 and 69.9%, respectively. The prepared SP-SeNPS efficiently suppressed the tested fungi growth with MIC (350 µg/ml) and MFCs (480–950 µg/ml). Most notably, biogenic SeNPs effectively extended the clot formation period recording 170.4 S for prothrombin time (PT) and 195.6 S for the activated partial thromboplastin time (aPTT). SP-SeNPs reduced the cell viability of breast adenocarcinoma (MCF-7) and ovarian cancer (SKOV-3) cell lines with a percentage of 17.6009% and 14.9484% at a concentration of 100 ug/ml, respectively. Moreover, SP-SeNPs could effectively alleviate the inflammation in RAW 264.7 macrophages with a reduction percentage of 8.82% in Nitric oxide concentration.

**Conclusion:**

The investigation findings reveal that SP-SeNPs are a hopeful antimicrobial, anti-tumor, anticoagulant, antioxidant, and anti-inflammatory factor that can be applied in medical cures.

## Introduction

Nanotechnology is a dynamic science and technology field that links various disciplines; physics, biology, chemistry, informatics, medicine, and engineering [1]. It deals with various matter structures having dimensions of a billionth of a meter [2]. Nanotechnology applications are numerous and include medicine, agriculture, industry, electronics, and biotechnology [3]. Nanoparticles are promising in several medical domains for many reasons such as their fascinating small size, highly reactive surface, bioactivity, modification capacity, and visual features [4]. There are various nanomaterials, including carbon-based ones, which are made mostly of carbon and can take the shape of tubes, hollow spheres, or ellipsoids. Nano silver, nanogold, quantum dots, and nano-oxides such as titanium dioxide are examples of metal-based nanomaterials. Composites integrate nanoparticles with larger, bulkier materials or with other nanoparticles. Denanmers are nanosized polymers composed of branching components [5]. Green synthesis is more advantageous than elderly synthesis techniques because it costs less by up to 40%, increases the production yield by 50%, and causes a 30% decline in energy expenditure [6].

The application of nanotechnology to regulate microbial growth—particularly multidrug-resistant ones has grown [1]. Antimicrobial-resistant bacteria and fungi are an essential concern that has become a complex global problem. Over 300 million serious fungal infections were reported worldwide, of which more than a million are deadly. The challenge and elevated expenses associated with addressing this issue, along with a rise in the fatality and morbidity rates of those impacted, might have detrimental effects on the healthcare and economic sectors [8, 9]. So, manufacturing novel treatments using natural nanotechnology chemicals, like nanoparticles of biological origin from selenium, gold, and silver with antimicrobial activity, is a fantastic alternative to compact this problem [10–12]. Selenium nanoparticles, or SeNPs, have recently piqued the attention of numerous researchers due to their low toxicity and bioavailability. Because of its increased bioactivity, selenium nanoparticles are commonly applied in biomedical applications and wastewater bioremediation [13, 14]. Selenium nanoparticles can be produced using chemical, biological, and physical methods. Nevertheless, organically synthesized SeNPS are favored for therapeutic purposes due to their greater compatibility with human tissues and organs. Many studies have investigated how their size, shape, and manufacturing process affect their use in biological systems [15].

Kong et al. [16] found that although it has been observed that selenium nanoparticles are safer than other selenium compounds such as sodium selenite, monomethylated selenium, and selenium methionine its intrinsic processes and anticancer potential are yet unknown. Over the past several decades, green nanomaterials have significantly advanced in many pertinent research disciplines thanks to their unique biological and physicochemical characteristics [17]. Therefore, nanoparticles have been employed extensively recently to treat microbial infections as a novel nonantibiotic therapeutic approach [18]. Nano selenium form is safer, inexpensive, and operative to be applied as an antimicrobial, antioxidant, and anti-inflammatory agent in biomedical remedies [19, 20] as the actual small particle size of SeNPs makes them easy to absorb through the human gastrointestinal tract [21]. Various precursors could create nanoparticles because of their intended applications. Utilizing living organisms for methods has several advantages, such as being affordable, readily available, environmentally safe, and non-toxic. Instead of using traditional stabilizing and reducing agents, biomolecules were employed [22]. Because they are composed of various organic compounds that act as powerful agents for reducing target metals, algae are an essential resource for producing nanoparticles [23]. One term for these algae might be “bionanofactories” containing numerous beneficial biological components that have made them an efficient tool for producing stable nanoparticles [24, 25].

Algae are essential for producing nanoparticles compared to other creatures like yeast, bacteria, and fungi [26]. The high stability of algae biosynthesized nanoparticles is due to the presence of numerous dynamic biological compounds in algal extracts, such as proteins, alkaloids, amines, phenolics, and pigments [27].

El-Sheek et al. [28] utilized *Spirulina platensis* and *Oscillatoria* sp. for the green synthesis of Ag_2_O|Ag-NPs and Au-NPs, respectively. Recently, *Spirulina platensis* extract was used in the sustainable synthesis of AgNPs to degrade brilliant blue dye [29]. Our study objectives are the phyco-synthesis and characterization of SeNPs using *S*. *platensis* extract and determining its antioxidant efficiency using the DPPH, ABTS, and H_2_O_2_ radical scavenging test. Determine its influence as an antimicrobial and antibiofilm agent against the selected microorganisms using the microtiter plate method. Evaluate its anticoagulant properties by measuring prothrombin time (PT) and the activated partial thromboplastin time (aPTT). Determine its antitumor efficiency against ovarian cancer and breast adenocarcinoma cell lines. Verify the anti-inflammatory properties of the bio-synthesized SeNPs by measuring the amount of nitric oxide.

## Materials and methods

### Chemicals and reagents

To construct the appropriate medium, all chemicals and solvents were acquired from Sigma Chemicals in analytical grade. The sodium selenite (Na_2_SeO_3_) supplier was Merck Chemicals Co. (Darmstadt, Germany).

### Cultivation of *Spirulina platensis*

The Freshwater Hydrobiology Laboratory, National Institute of Oceanography and Fisheries (NIOF), Cairo, Egypt, provided the cyanobacterium *S*. *platensis* used in this work. The strain was kept in 500 mL sterilized Erlenmeyer flasks with 100 mL of Zarrouk's medium [30] at 30 ± 2 °C and pH 9 with manual shaking two times a day and continuous lighting with cool white, a fluorescent lamp's light intensity of 50 µmol photons/m^2^/s. The distance between the flask and the light source was maintained at [12 inches].

### Preparation of algal extract

In a water bath, 50 mL of Milli-Q-water and 500 mg of lyophilized algal biomass were incubated at 90° C for one hour. The sample was then sonicated for one minute. During sonication, the sample was kept in an ice bath; after that, centrifugation was performed for 10 min at 5000 rpm. The nanoparticles were then subjected to a washing step with dist. water by centrifuging at the same RPM and duration to remove any residual contaminants or unbound substances. The washing procedure was repeated three times to ensure that the nanoparticles were thoroughly cleaned to produce a cell-free and aqueous extract (1% solution). A cell-free extract is contained in the supernatant that is produced following centrifugation. Until it is needed, it is stored at 4 ºC after being filtered through Whatman No. 1 filter paper.

### Synthesis of selenium nanoparticles and purification

**Fig. 1:**
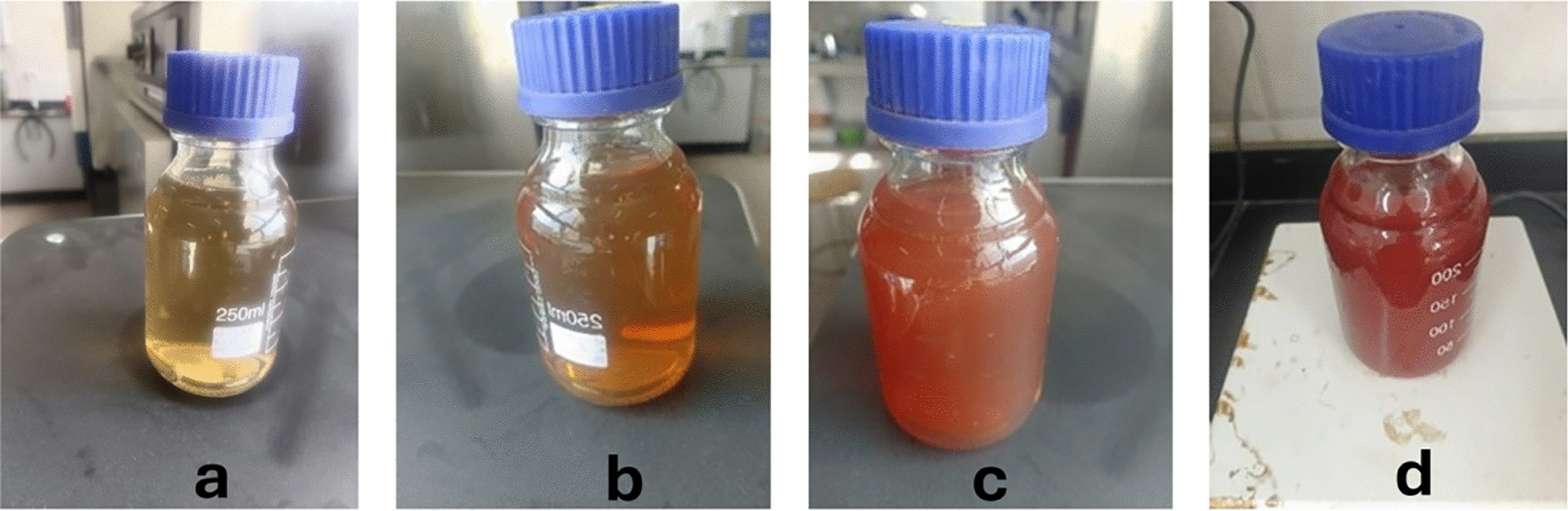
Biosynthesis of SeNPs with *Spirulina platensis* extract. **a** SP-SeNPs on day 1. **b** SP-SeNPs on day 2. **c** SP-SeNPs on day 4. **d** SP-SeNPs on day 6

Different concentrations (1 mM) of sodium selenite (Na_2_SeO_3_) were added to 2% *S. platensis* extract in a flask holding 50 ml of Milli-Q water to synthesize selenium nanoparticles. Under dark conditions, the reaction mixture was incubated at 130 rpm and 37 °C on an orbital shaker. As seen in Fig. 1, the reaction mixture's color evolved from green to ruby red to crimson after being incubated for 6 days. The color change indicates the formation of selenium nanoparticles. Following the color shift, centrifugation is used to purify selenium NPs as illustrated in Fig. 1.

### Characterization of bio-transformed selenium nanostructures

#### UV–visible spectroscopy

To provide additional characterization, the reaction mixture's corresponding surface plasmon resonance (SPR) peaks were examined in UV–visible spectroscopy. The UV-Vis absorption spectra were obtained on Cary series UV-Vis—NIR, Australia, to record the UV-visible spectrum in the 200–800 nm range.

#### Fourier transform infra-red (FTIR) analysis

The possible biomolecule active groups that control the capping and reduction of nanoparticles were found using FTIR. The Chemistry Laboratory at South Valley University, Egypt, used potassium bromide (KBr) as a test material in the 450–4000 cm^−1^ wave range for FTIR spectrum analysis. Plotting the resulting peaks showed transmittance (percent) on the X-axis and wave number (cm^−1^) on the Y-axis.

#### Dynamic light scattering and zeta-potential measurement

Zeta potential, a measurement of the charges on the nanoparticles' surface. The stability of the nanoparticles will also be shown by the zeta potential values, which were calculated based on the charged particles behaving in an electric field. Zeta-sizer Nanez's tools were used to measure them. Using a Zeta-sizer Nano ZS Particle Analyzer (Malvern, UK), synthetic selenium nanoparticles' size, distribution, and zeta potential were determined. The zeta potential was recorded in the –200 to + 200 mV range.

### Transmission electron microscopy (TEM)

Talos F200i (Thermo Scientific, USA) high-resolution transmission electron microscope was run TEM at 200 kV accelerating voltage. Using a droplet of colloid suspension in the corresponding solvent on a Formvar carbon-coated, 300-mesh copper grid (Ted Pella), a sample for TEM was made and permitted to evaporate in the air at normal conditions.

### X-ray diffraction (XRD)

An XRD pattern has been performed using the XPERT-PRO Powder Diffractometer system, with 2 θ (10°—80°), with minimum step size 2θ: 0.001, and at a wavelength (Kα) = 1.54614 °[31].

### Antioxidant properties of Se-NPs by DPPH radical scavenging assay

Trolox Standard for DPPH assay was prepared with a concentration of 2 mM. This stock solution prepared the following final concentrations in methanol: 100, 80, 60, 40, 30, and 15 μM. The sample concentration was 2 mg/mL. The 2,2-diphenyl-1-picryl-hydrazyl-hydrate (DPPH) free radical assay was conducted using the methodology of [32], incorporating the adjustments suggested by Elkhloly et al. [33]. In summary, 100 μL of newly made DPPH reagent (0.1% in methanol) was combined with 100 μL of the sample in a 96-well plate (n = 3). The reaction was then allowed to sit at room temperature for 30 min without light. At the end of the incubation period, the subsequent drop in DPPH color intensity was recorded at 540 nm. Equation % inhibition = (Average absorbance of blank-average absorbance of the test)/ (Average absorbance of blank)) * 100 represents data as means ± SD. A Fluster Omega microplate reader was used to record the results. The linear regression equation derived from the following calibration curve (the linear dose–response curve of Trolox) displays the samples' ability to decrease DPPH as μl TE/mg sample.

### Antioxidant properties of Se-NPs by ABTS radical scavenging assay

The produced SP-SeNPs were tested for antioxidant activity using the ABTS radical scavenging technique [34, 35]. The reaction of ABTS stock solution (1.8 mM) (Sigma, PN: A3219) with 0.63 mM potassium persulfate at ambient temperature for 16 h in the dark produced ABTS radical cation (ABTS +). Next, the solution was diluted with ethanol until absorbance reached 0.700 (± 0.030) at 734 nm. At ambient temperature, measurements were taken. Various sample concentrations were diluted with 80% methanol. Later, 190 μl radical solution and 10 μl diluted extracts were combined in a microtiter plate. After mixing, 734 nm absorbance was measured every 1 min for 13 min. Each assay's solvent blanks were appropriate. The usual antioxidant and negative controls were BHT and methanol (80%). Three replicates of each sample were used in the experiments. Using Graphpad Prism (San Diego, CA, USA), graphic plots of the dose–response curve were used to estimate the 50% inhibitory concentration (IC50) for 50% radical scavenging activity.

### Hydrogen peroxide (H_2_O_2_) scavenging capacity

Hydrogen peroxide was determined following the method of [36]. A solution of hydrogen peroxide (40 mM) was prepared in phosphate buffer (pH 7.4). SP-SeNPs (100 μg/mL) were added to a hydrogen peroxide solution (0.6 mL, 40 mM). The absorbance of hydrogen peroxide at 230 nm was determined 10 min later against a blank solution containing the phosphate buffer without hydrogen peroxide. The percentage of hydrogen peroxide scavenging of SP-SeNPs extract and standard compounds.

Were calculated: % Scavenged [H_2_O_2_] = [(AC – AS)/AC] × 100.

Where AC is the absorbance of the control and AS is the absorbance in the presence of the sample of test or standards.

### Antibacterial efficacy of *S*. *platensis*, sodium selenite, and Se-NPs

The antibacterial efficacy was evaluated against 13 indicator strains. They were obtained kindly from the International Luxor Hospital and bacteriology laboratory, Faculty of Science, South Valley University. The tested strains were* Escherichia coli*, *Proteus vulgaris, Klebsiella pneumoniae*, *Pseudomonas cepacia*, *Pseudomonas fragi*, *Enterobacter cloace*, *Enterobater aerogenes*, *Serratia liquifaciens*, *Staphylococcus epidermidis*, *Staphylococcus aureus, Streptococcus pyrogenes*, *Enterococcus faecalis,* and *Bacillus subtilis*. Minimum inhibitory concentration (MIC) and minimum bactericidal concentration (MBC) of *S. platensis*, sodium selenite, and Se-NPs were ascertained by use of the broth-dilution microtiter technique and INT (*p*-iodonitrotetrazolium violet chloride) formazan assay in 96 well plates. Using a multichannel pipette, MIC was garnered by inoculating 100 µl of indicator organisms (OD _595_ 0.001) into a series of wells with different volumes (10–100 µl) of *S. platensis*, sodium selenite, and the synthesized Se-NPs. *S. platensis*, sodium selenite, and the green synthesized Se-NPs were then moved and mixed from columns 1–10. Column 11 is considered as a standard well containing 100 μl of culture media without bacteria, *S. platensis*, sodium selenite, and Se-NPs. Finally, column 12 was a positive control containing 100 μl of culture media bacteria without *S. platensis*, sodium selenite, and Se-NPs. The MIC is the lowest dosage of antibacterial agent that, following a regulated incubation, visually inhibits the color change of INT [37, 38]. After the MIC determination, aliquots of 0.01 ml from all wells, which showed no visible bacterial growth (prevents colour change of INT), were seeded in tryptic soya agar plates (TSA). Now, any organisms that the MIC test suppressed but did not kill have an opportunity to proliferate because *S. platensis*, sodium selenite, and Se-NPs have been diluted significantly. The lowest antibacterial agent concentration that has reduced the number of colonies by 99.9% following a typical incubation period is known as MBC [39].

### Antibiofilm assay

The crystal violet method assessed the antibiofilm activity of *S. platensis*, sodium selenite, and Se-NPs. Briefly, *Escherichia coli*, *Klebsiella pneumoniae*, *Proteus vulgaris*, *Pseudomonas cepacia*, *Pseudomonas fragi*, *Enterobacter cloace*, *Enterobacter aerogenes*, *Serratia liquifaciens*, *Staphylococcus aureus*, *Staphylococcus epidermidis*, *Streptococcus pyrogenes*,* Bacillus subtilis* and *Enterococcus fecalis* were cultured in TSA medium (Oxoid) for 24 h at 37 °C. OD_595_ of 0.02 were prepared using tryptic soya broth (TSB). Each well of a 96-well plate was filled with 130 µl of prepared culture, incubated for 24 h at 37 °C and inoculated with 50 µl of *S. platensis*, sodium selenite, and Se-NPs and re-incubated again for 24 h at 37 °C. Column containg130 µl of prepared culture and incubated at 37 °C for 24 h served as control. To get rid of any free-floating bacterial strains, each well was then carefully removed and cleaned six times with sterile distilled water. Staining with 160 µl of 1% (wt/vol) crystal violet solution was used to assess the tested strains' adherence to the 96-well plate. After being washed four more times to get rid of any remaining stain, the 96-well plate was left to dry. Lastly, 210 µl of 95% ethanol was used for 45 min to destain the biofilm mass. Infinite^®^ F50 Robotic (Ostrich) Microplate Reader was employed to determine the biofilm formation's OD value concerning crystal violet at 600 nm. OD values of samples with *S. platensis*, sodium selenite, and Se-NPs were compared with the control sample [40–42]**.**

### Antifungal efficiency of *S*. *platensis*, sodium selenite, and phyco-synthesized Se-NPs extracts

Microtiter plate technique was applied to determine the antifungal activity of *S*. *platensis*, sodium selenite, and phyco-synthesized Se-NPs extracts against the growth of *Candida albicans* MH534933**,** *C. glabrata* MH534928**,** *Alternaria cerealis* MT808477 **(**obtained from Assiut University Mycological Center (AUMC**)**, and *Aspergillus flavipes* ON644533 (obtained from mycology laboratory, South Valley University)**.** The tested fungi were subcultured on potato dextrose agar (PDA) medium.

### Inoculum preparations

Spore suspension of the tested *Candida* was adjusted to 0.5 McFarland by adding an inoculum of cultured *Candida* in 0.9% saline. Then, 9.5 ml of potato dextrose broth was added to 0.5 ml of the adjusted 0.5 McFarland suspension, and well-shaken to obtain 1.8 × 10^5^ CFU/ml. A hemocytometer adjusted the spore suspension of *A. flavipes* ON644533, and *A*. *cerealis* MT808477 to (2 × 10^6^ CFU/ml) [43].

### Determination of the minimum inhibitory concentration (MIC) and the minimum fungicidal concentration (MFC)

The method described by Suurbaar et al. [44] with slight modification was applied, 100 μl of potato dextrose broth (PDB) was added to each well of 96 sterilized microtiter plates, different volumes (10, 20, 40, 50, 100, and 150 µL) of *S*. *platensis*, sodium selenite, and phyco-synthesized Se-NPs extracts were added. 5 μl of adjusted fungal suspension was added to each well. Nystatin concentrations of 17.5, 8.8, and 4.4 mg/ml were used as a positive control, and media without fungal suspension or tested *S*. *platensis*, sodium selenite, or phyco-synthesized Se-NPs was considered a negative control. The mixture was mixed well, and then, the 96 microtiter plates were incubated at 30–35 °C for 24 h for tested *Candida* and 3 days for tested filamentous fungi at 30 °C [45] in a shaker incubator at 150 rpm. After that, all wells were supplemented with 5 μl of 0.01% resazurin dye and incubated for 2 h in a shaker incubator (150 rpm) at 35–37 °C. The blue color of resazurin indicates fungal inhibition and the lowest inhibition volume was recorded as MIC, but the change to pink or colorless indicates fungal growth [46]. MFC was evaluated by culturing 10 µL of MIC wells and the higher concentrations on PDA medium and incubation at 37 °C for 24 h for tested *Candida* and 28 ^o^C for 48 h for tested filamentous fungi. The absence of visible growth in the petri dish is regarded as MFC [47].

### Anticoagulant properties

In vitro, the anticoagulant activity of *S. platensis*, sodium selenite, and Se-NPs was examined in the blood samples by measuring prothrombin time (PT) and the activated partial thromboplastin time (aPTT) [48, 49]. Using sterile syringes, vein punctures were performed on healthy participants to obtain approximately 6 ml of blood. To prevent natural coagulation, blood was drawn in a sodium citrate test tube (9:1 v/v). Centrifugation was started right away and ran for 15 min at 3000 rpm. Following centrifugation, plasma was gathered, and blood cells were disposed of. For PT determination, 90 µL of plasma and 10 µL of *S. platensis*, sodium selenite, and Se-NPs were mixed. Two controls were used: blood plasma of healthy volunteers with and without heparin. The mixture was first incubated at 37 °C for 5 min. then 200 µL of PT assay reagent **(SIEMENS)** was added. The clotting time is calculated as soon as the PT reagent is added using **CoaDATA 504 (Germany).**

Before the mixture was incubated for three minutes at 37 °C, 50 µL of pre-warmed aPTT assay reagent (HIGH TOP) was completely mixed. Then, add 50 µL of prewarmed 0.025 mol/L CaCl_2_ to induce clotting **(HIGH TOP)**, the time of clotting is calculated as soon as adding CaCl_2_ using **CoaDATA 504 (Germany)** and compare heparin clotting time.

### *In-vitro* cytotoxicity evaluation of Se-NPs on SKOV-3 and MCF-7

*Cell culture* SKOV-3: Ovarian cancer and MCF-7: Breast Adenocarcinoma were obtained from Nawah Scientific Inc., (Mokatam, Cairo, Egypt). SKOV-3 cells were kept in RPMI and MCF-7 in DMEM medium supplemented with 10% heat-inactivated fetal bovine serum, 100 mg/mL streptomycin, and 100 units/mL penicillin in a humidified, 5% (v/v) CO_2_ environment at 37 °C.

*Cytotoxicity assay* The SRB assay was used to measure cell viability according to [50, 51].

### *In-vitro* anti-inflammatory assay by quick screening and SRB

*Cell culture* Nawah Scientific Inc. (Mokatam, Cairo, Egypt) provided the RAW 264.7 mouse macrophage cell line. Cells were kept in DMEM medium at 37 °C in a humidified, 5% (v/v) CO_2_ atmosphere supplemented with 100 mg/mL streptomycin, 100 unit's/mL penicillin, and 10% heat-inactivated fetal bovine serum.

*Quick Screening* A 96-well plate was seeded with RAW264.7 cells and incubated for 24 h. The following day, untreated cells were replaced with fresh medium (Control group) and inflammation was triggered with 1 μg/mL of LPS (LPS-group). LPS will be applied to compounds at two or five concentrations (LPS + Drug). Dexamethasone (1uM) served as a positive control for anti-inflammation. The amount of nitric oxide (NO) secreted was measured by mixing equal amounts of Griess reagent and the cell supernatant at room temperature for 10 min in the dark. Nitrite concentration was measured using an ELISA plate reader as absorbance at 540 nm [52, 53].

*SRB Screening* was determined following the techniques mentioned by [50, 51].

## Results

Using an aqueous *Spirulina platensis* extract as a bio-reductant to decrease sodium selenite, selenium nanoparticle synthesis and purification were accomplished satisfactorily **(**Fig. 1**)**. The Na_2_SeO_3_ solution was colorless at first. After adding *S. platensis* cell-free extract, the reaction mixture turned light green. After 24 h, the mixture turned orange-red, indicating the development of SP-SeNPs **(**Fig. 1**)**.

### Characterization UV–visible spectroscopy

**Fig. 2:**
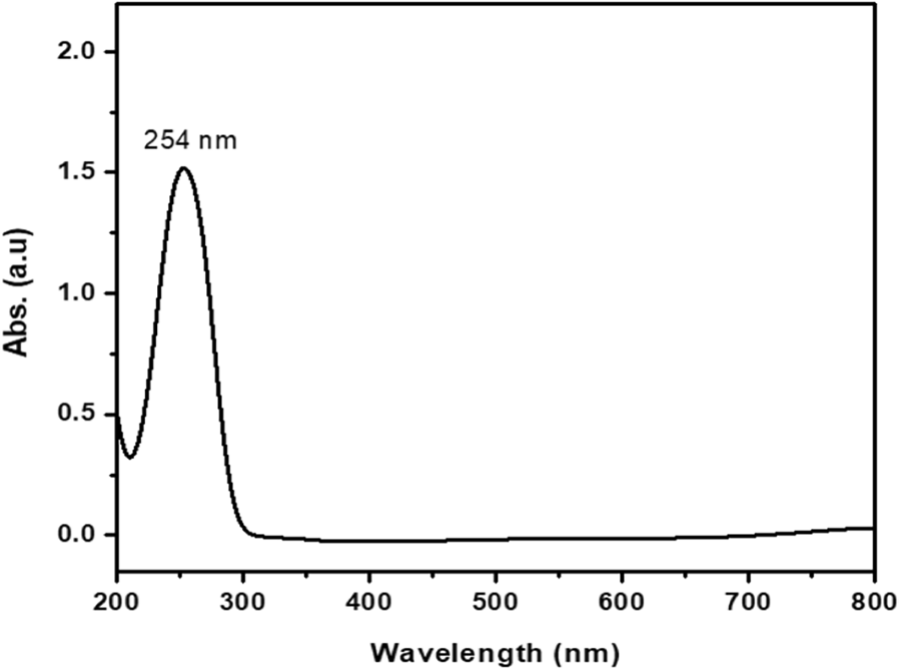
Shows the optical absorption of the prepared selenium nanoparticles

Various analytical methods have been utilized to characterize the phyco-synthesized selenium nanoparticles, which have been extensively researched. The process primarily initiates with UV–visible spectroscopy. After incubation, the reaction mixture transitioned from green to red, indicating selenite reduction and selenium nanoparticles' formation. UV–visible spectroscopy further corroborates this, with the samples exhibiting the corresponding absorption maxima in the 254 nm region, as illustrated in Fig. 2.

### FTIR analysis

**Fig. 3:**
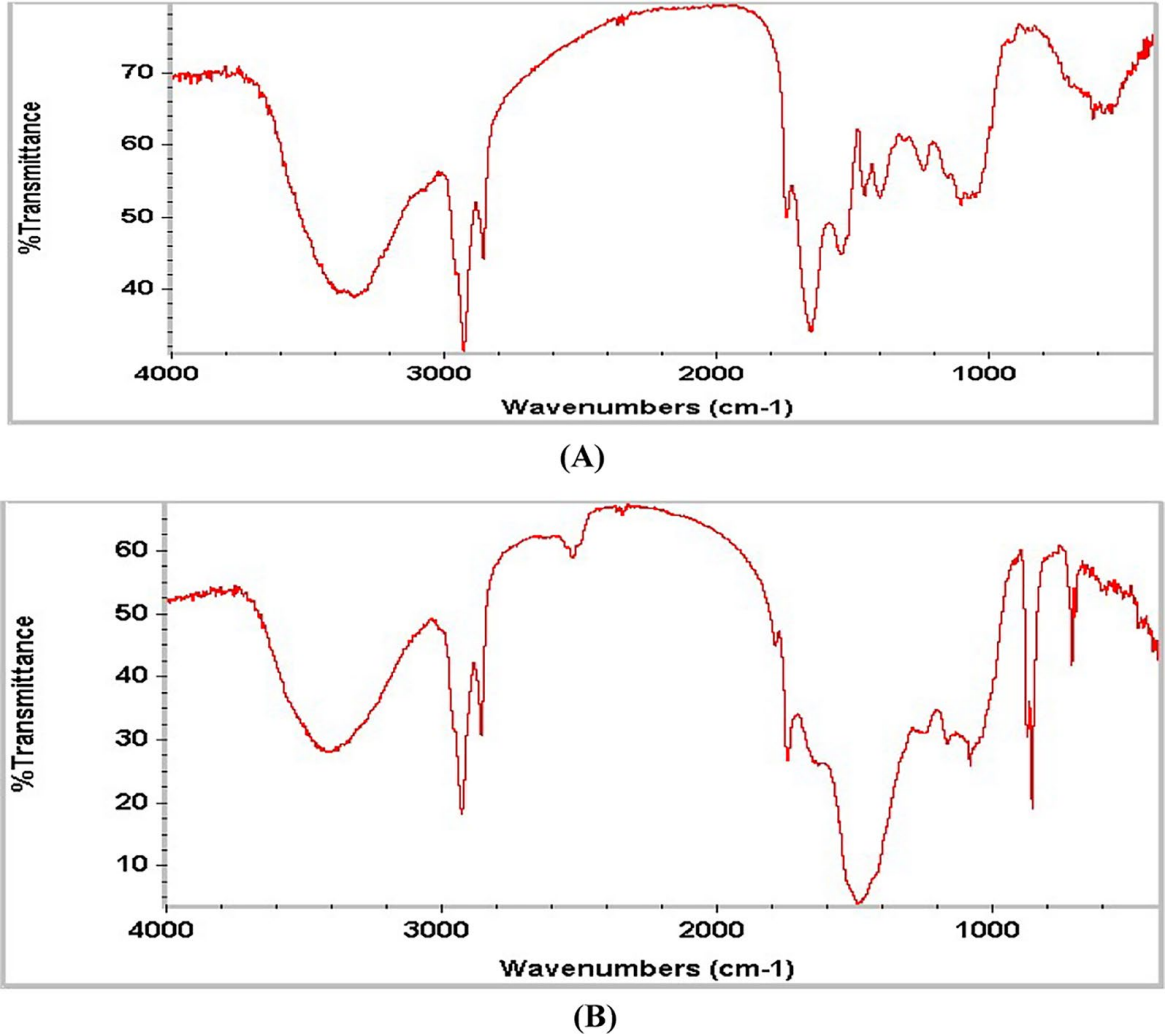
Fourier transform-infrared spectrum of (**A**) *S. platensis* extract and (**B**) *S. platensis* selenium nanoparticles (SP-SeNPS)

FTIR spectroscopy was used to detect the functional groups on *Spirulina* selenium nanoparticles surface. The algal cell extract and *Spirulina*-selenium nanoparticle FTIR spectra are shown in Fig. 3.

FTIR was used to discover *S*. *platensis* biomolecules that decrease and stabilize selenium ions. The FT-IR spectra of *Spirulina platensis* derived green SeNPs show absorption peaks at 3527.85, 2826.05, 2728.56, 1085.34, 1275.44, and 671.41 cm^−1^. In Fig. 3, the Peak at 3527.85 represents functional groups like OH stretching of alcohols or phenols, N–H groups (amines peptide chain and proteins). Wavenumbers 2826.05 and 2728.56 cm^−1^ indicate the presence of aliphatic bond (C-H) in proteins and lipids. Vibration peaks at 1085.34 and 1275.44 cm^−1^ confirm the presence of ether and carboxyl group (C = O), respectively. The vibration peak at 671.41 cm^−1^ represents C–H in aromatic compounds. According to FTIR spectroscopy, selenium nanoparticle bio-reduction eliminates secondary metabolites by reducing selenium ions by polyols, which oxidize to generate unsaturated carbonyl groups.

### Dynamic light scattering (DLS)

**Fig. 4:**
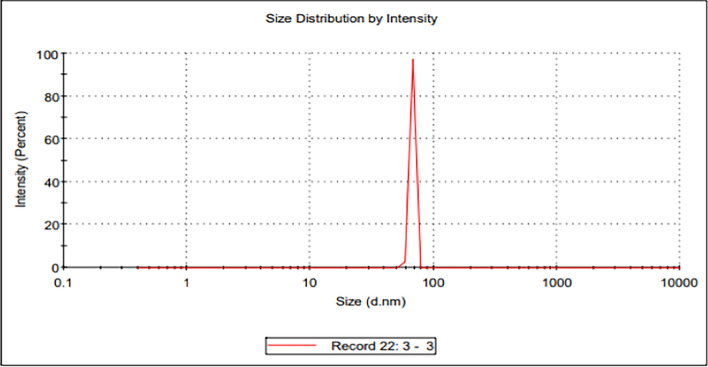
Dynamic light scattering analysis of *Spirulina* selenium nanoparticles

The scientific method for determining the size distribution profile of small particles in suspension or polymers in solution is dynamic light scattering, also called quasi-elastic light scattering or photon correlation spectroscopy. Data in Fig. 4 shows the population or size distribution of selenium nanoparticles produced by *S*. *platensis* using the Malvern instrument's Dynamic Light Scattering method. Using DLS and zeta potential measurement, the hydrodynamic size distribution and stability of the SP-SeNPs were ascertained. With a mean hydrodynamic size of 411.3 ± 8.529 nm (Z-average) and a polydispersity index (PDI) of 0.548 ± 0.021, the DLS pattern showed a single broad peak (Fig. 4), and the core size equals 134 nm, suggesting polydispersity in the nanoparticles' size distribution.

### Transmission electron microscope (TEM)

**Fig. 5:**
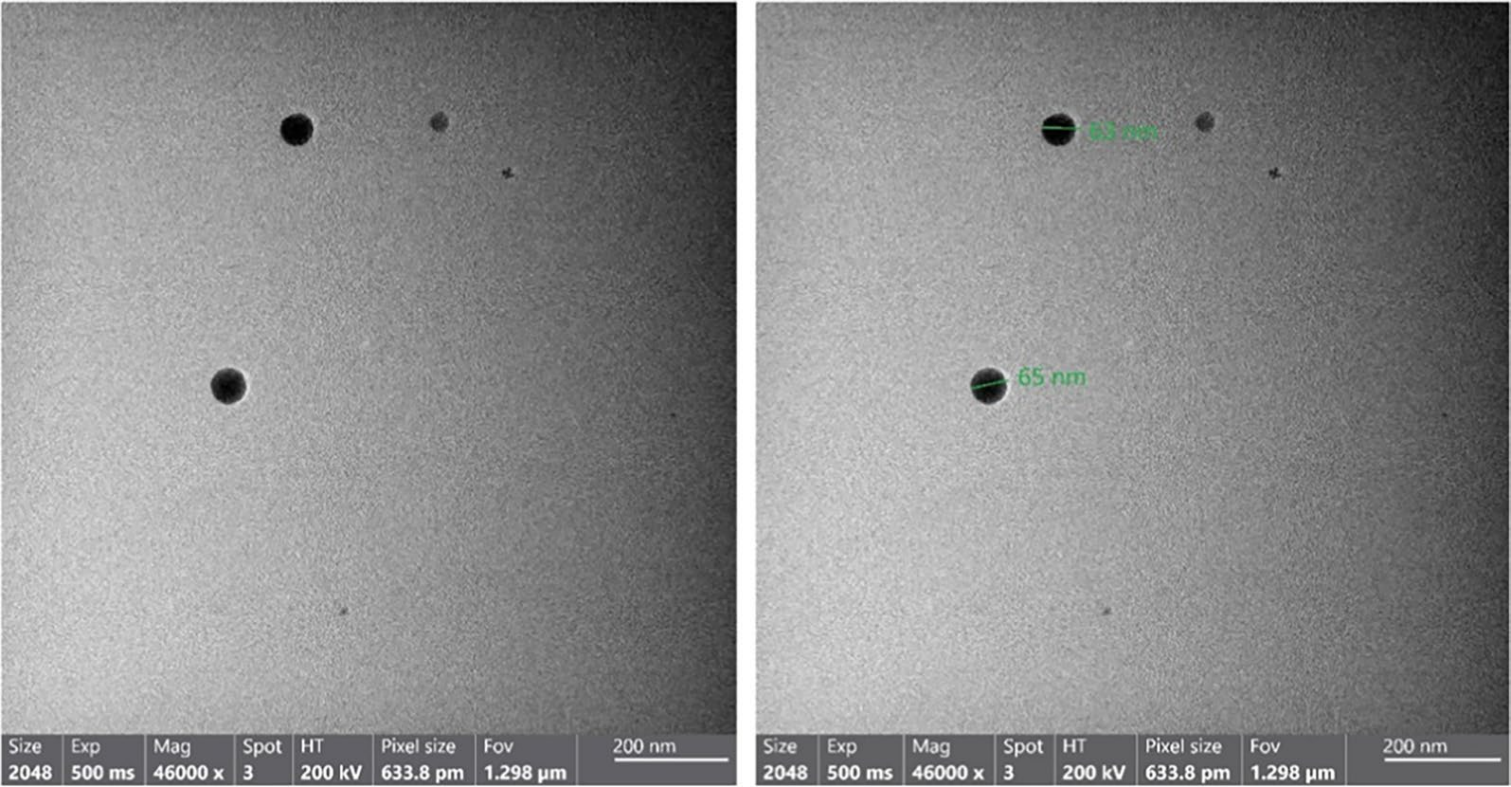
TEM images of the prepared SP-SeNPs showing the particle size

Permitting to TEM micrographs, the biosynthesized SP-SeNPs were smooth-surfaced spheres with a 65 nm diameter (Fig. 5).

### X-ray diffraction (XRD) analysis

**Fig. 6:**
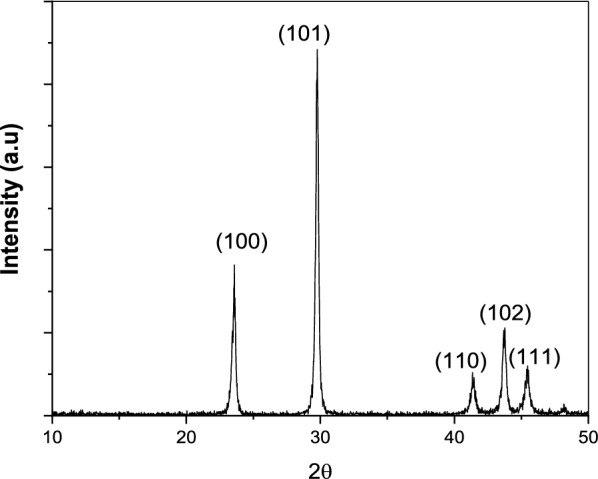
XRD analysis of SP-SeNPs

The nature of the crystalline phase and the size of Se are determined by using X-ray diffraction. The XRD patterns obtained show the main peaks characteristic of crystalline-Se at 2θ values of 23.5°, 29.73°, 41.4°, 43.68°, and 45.39° (Fig. 6), corresponding to the crystal planes (100), (101), (110), (102), and (111), respectively. Reflections of the pure hexagonal phase of selenium crystals with lattice parameters a = 4.368 Å, b = 4.368 Å, and c = 4.9536 Å (JCPDS card No. 01-086-2246). The average crystalline size of Se nanostructures measured by Scherrer's equation was about 31.74 nm, Crystallite size values are in the nano-scale, confirming the nanostructure properties for Se

$$D=\frac{{K}_{S}\lambda }{\beta\, \mathit\,{cos}\,\theta }$$

### Antioxidant properties of SP-SeNPs by DPPH radical scavenging assay

**Fig. 7:**
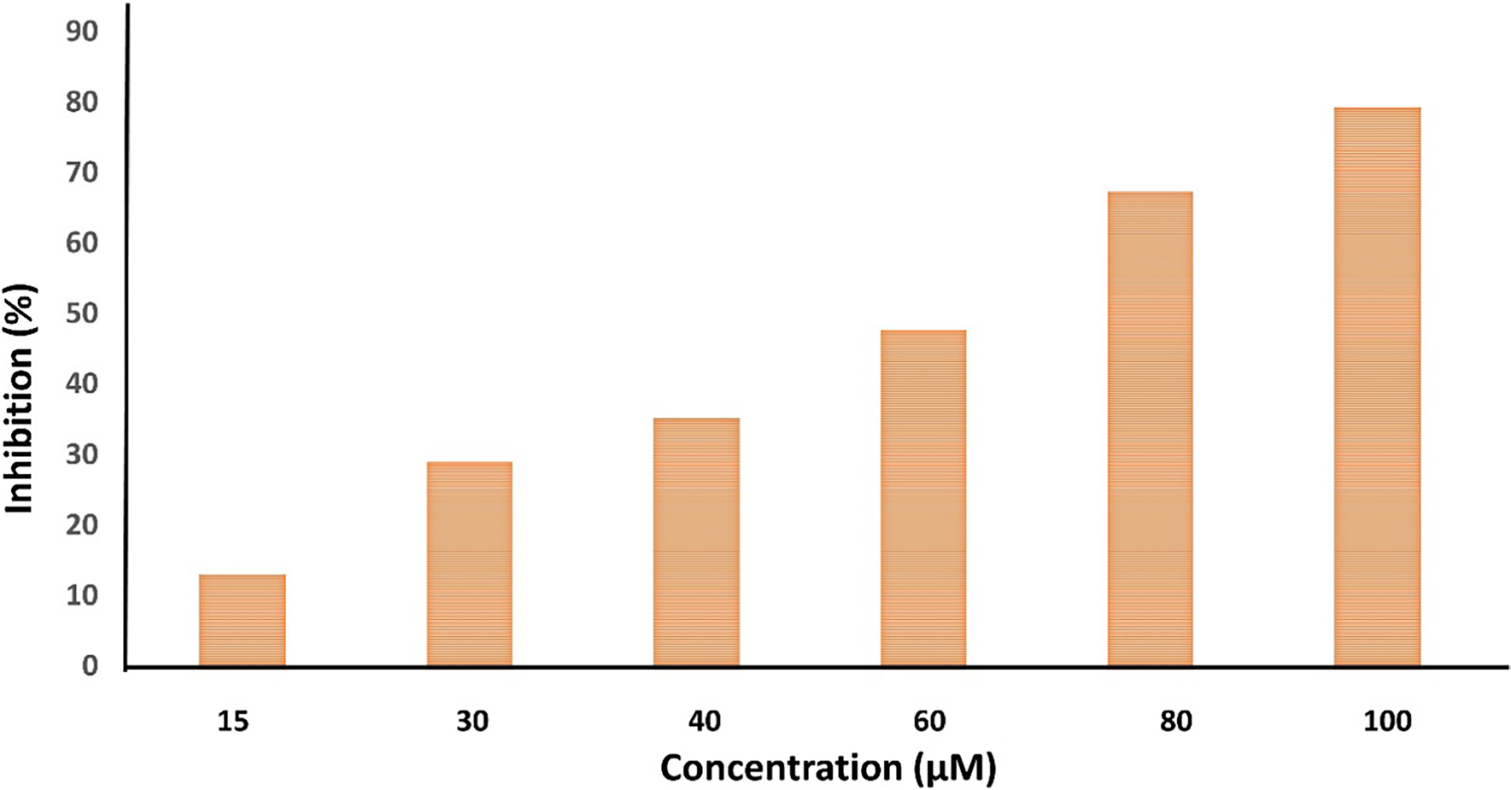
DPPH inhibition (antioxidant activity) of SP-SeNPS

Significant antioxidant properties of the synthesized Se-NPs. Synthetic Se nanoparticles can donate electrons to the stable molecule 2,2-diphenyl-1-picrylhydrazyl (DPPH). The antioxidant effectiveness of Se-NPs was assessed through spectrophotometry, which involved the conversion of the purple DPPH color to yellow. As the concentration of the produced Se-NPs samples increased, there was a corresponding rise in DPPH radical scavenging. The data illustrated in Fig. 7 indicates that the percentage of inhibition rose from 13.073% to 79.234% as the concentration increased from 15 to 100 µM. The most significant absorption occurs at 517 nm (purple) during the interaction between free-radical DPPH and an unpaired electron. DPPHH exhibits lower absorbance than DPPH because of the reduced hydrogen content, formed when an antioxidant free-radical scavenger interacts with DPPH. As additional electrons accumulate, this radical form undergoes decolorization, resulting in a yellow hue, in contrast to the DPPH-H state. The present study observed that the scavenging of DPPH radicals increased with the rising concentration of the synthesized Se-NPs samples (Fig. 7).

### Antioxidant properties of SP-SeNPs by ABTS and H_2_O_2_ scavenging assay

SP-SeNPs showed potent antioxidant activity by ABTS and H_2_O_2_ radical scavenging assay, and the scavenging percentage is concentration-dependent (Table 1). The concentration of SP-SeNPs needed to inhibit 50% of the free radicals (IC50) was 50.69 and 116.18 µg/ml for ABTS and H_2_O_2_ radical scavenging activity, respectively. However, IC50 was found to be 10.66 and 14.77 µg/ml for ABTS and H_2_O_2_ radical scavenging assay in case of using ascorbic acid.

**Table 1:** The antioxidant potency of SP-SeNPs and ascorbic acid by ABTS and H_2_O_2_ radical scavenging assay

Sample conc. (µg/ml)	Ascorbic acid ABTS scavenging %	SP-SeNPs ABTS scavenging %	Ascorbic acid H_2_O_2_ scavenging %	SP-SeNPs H_2_O_2_ scavenging %
1000	95.46	82.21	95.73	80.48
500	95.30	70.21	92.55	72.49
250	93.05	61.03	89.91	64.53
125	91.29	52.40	83.72	52.99
62.5	85.22	41.62	73.42	31.52
31.25	72.28	33.30	63.30	19.28
15.6	62.36	25.27	51.79	11.97
7.8	42.39	18.85	34.70	6.17
3.9	33.10	11.89	25.36	3.03
2	24.98	5.35	18.43	1.39
1	18.13	2.20	11.73	0.81
0.5	10.87	1.05	6.20	0.34
0	0	0	0	0

### Antibacterial efficacy of Se-NPs, sodium selenite, and *S*. *platensis*

Results presented in Table 2 showed that no antibacterial efficacy was recorded for *S*. *platensis* alone at the used concentrations against all tested bacterial strains. Sodium selenite has antibacterial effect on *Pseudomonas cepacia*, *Pseudomonas fragi*, and *Serratia liquifaciens* with MIC of 444–500 µg/ml and MBC of 474–500 µg/ml. Interestingly, Se-NPs have antibacterial efficiency against *Escherichia coli*,* Proteus vulgaris*,* Pseudomonas cepacia*, *Pseudomonas fragi*, *Enterobacter cloace*, *Serratia liquifaciens*, and *Bacillus subtilis* with MIC of 230–333 µg/ml and MBC of 286–333 µg/ml.

**Table 2:** Sodium selenite and SeNPs' minimum inhibitory concentration (MIC) and minimum bactericidal concentration (MBC)

Parameters	MIC µg/ml		MBC µg/ml	
Tested strains	Se	Se-NPs	Se	Se-NPs
*E. coli*	− ve	286	− ve	333
*K. pneumoniae*	− ve	− ve	− ve	− ve
*P. vulgaris*	− ve	286	− ve	333
*P. cepacia*	500	230	500	286
*P. fragi*	444	230	474	286
*E. cloace*	− ve	230	− ve	286
*E. aerogenes*	− ve	− ve	− ve	− ve
*S. liquifaciens*	444	286	474	333
*S. aureus*	− ve	− ve	− ve	− ve
*S. epidermidis*	− ve	− ve	− ve	− ve
*S. pyrogenes*	− ve	− ve	− ve	− ve
*E. fecalis*	− ve	− ve	− ve	− ve
*B. subtilis*	− ve	333	− ve	333

− ve: negative effect at the tested concentration, Se: sodium selenite, Se-Nps: selenium nanoparticles, no effect has been detected for* Spirulina platensis* at the used concentration

### Antibiofilm activity

**Fig. 8:**
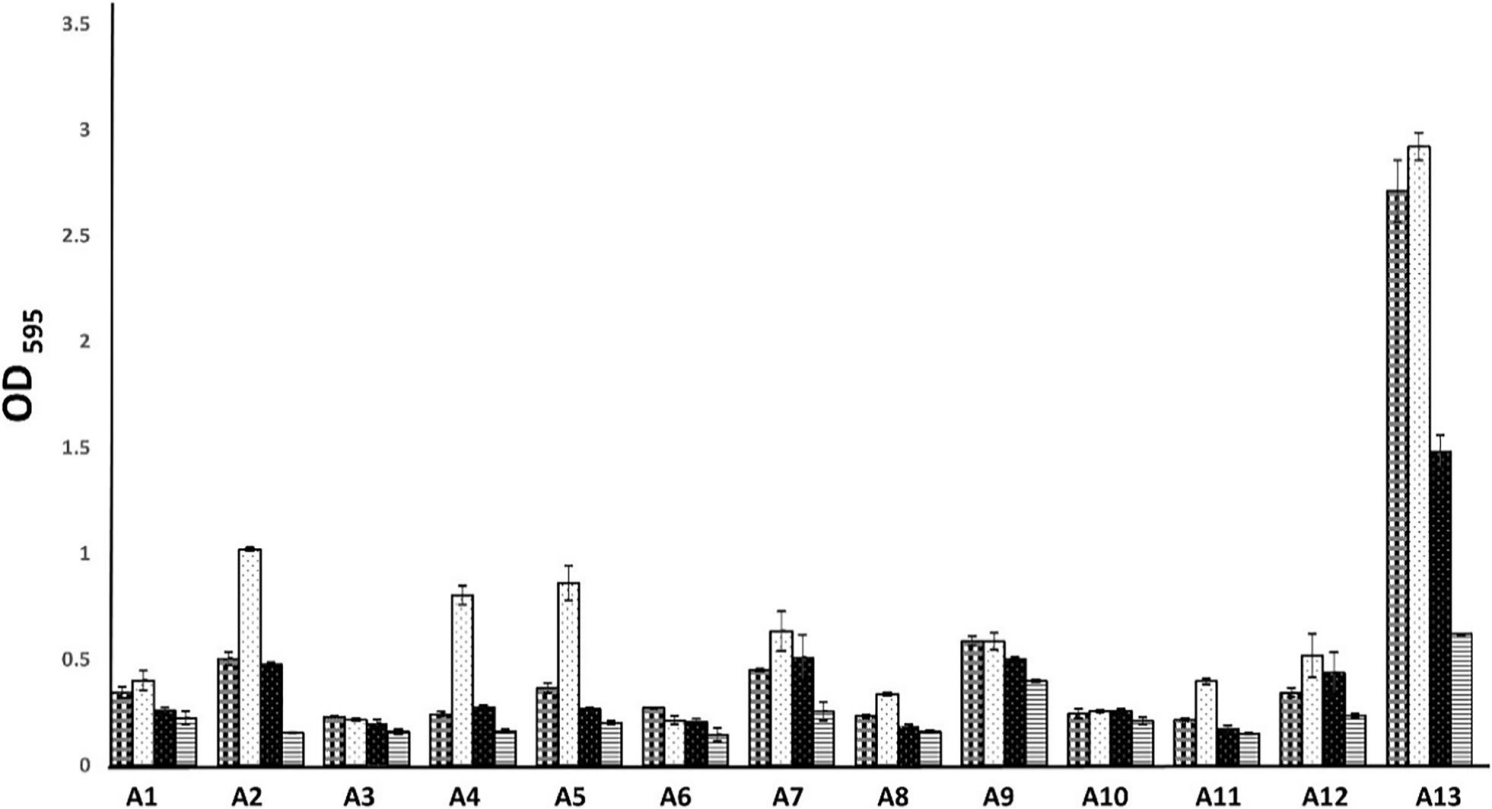
Antibiofilm activity of SeNPS; A1: *Escherichia coli*; A2: *Klebsiella pneumoniae*; A3: *Proteus vulgaris*; A4: *Pseudomonas cepacia*; A5: *Pseudomonas fragi*; A6: *Enterobacter cloace*; A7: *Enterobacter aerogenes*; A8: *Serratia liquifaciens*; A9: *Staphylococcus aureus*; A10: *Staphylococcus epidermidis*; A11: *Streptococcus pyrogenes*; A12: *Enterococcus fecalis*; A13: *Bacillus subtilis*

Antibiofilm efficacy of SeNPs was evaluated using crystal violet assay, the finding showing that *S*. *platensis* increased biofilm mass of all tested strains except in the case of *Enterobacter cloacae*, *Proteus vulgaris*, and *S. aureus* biofilm inhibited by 22.4, 6.1, and 0.3%, respectively. Sodium selenite has variable effects on biofilm formation of tested strains where it increases biofilm mass of *Pseudomonas cepacia*, *Enterobacter aerogenes*, *Staphylococcus epidermidis*, and *Enterococcus fecalis* while inhibiting the biofilm of the other tested strains and the highest percentage of inhibition was on the biofilm of *B. subtilis* (45.3%), and the lowest percentage was on biofilm of *Klebsiella pneumoniae* (5.5%). Synthesized selenium nanoparticles significantly inhibited the biofilm formation of all tested strains. The highest inhibition percentages were recorded for *B. subtilis* and *Klebsiella pneumoniae,* with 78.8 and 69.9%, respectively, followed by *Enterobacter cloace* and *Pseudomonas fragi* with 47.1 and 44.6% percentages. The lowest percentage of inhibition was recorded on *Streptococcus pyrogenes* and *Staphylococcus epidermidis,* with percentages of 30.7 and 14.4%, respectively, as demonstrated in (Fig. 8).

### Antifungal properties of *S*. *platensis*, sodium selenite, and phyco-synthesized Se-NPs

The shown results in Table 3 indicated that the fungal growth was inhibited by *S*. *platensis* extract, sodium selenite, and phyco-synthesized Se-NPs with variable efficiencies. *S*. *platensis* exhibited the lowest antifungal efficiency and did not show fungicidal activity against the tested fungal strains. *S*. *platensis* extract was effective only against *Candida albicans* MH534933 and *C. glabrata* MH534928 with MICs (9.5 µg/ml) and did not show any inhibitory effect against the growth of *Alternaria cerealis* MT808477 and *Aspergillus flavipes* ON644533. Sodium selenite has effectively suppressed the growth of the tested *Candida* and filamentous fungi with MICs (350 and 625 µg/ml), and MFCs (625 µg/ml) were observed in the tested fungi except *Alternaria cerealis* MT808477 were not repressed. Selenium nanoparticles were the most effective and showed fungistatic and fungicidal efficacies against all the tested fungi with MICs (350 µg/ml). The lowest MFC (480 µg/ml) was recorded against *C. albicans* MH534933. MFCs of 580 µg/ml were observed against *C. glabrata* MH534928 and *A. flavipes* ON644533*. Alternaria cerealis* MT808477 was completely repressed at concentration of 950 µg/ml. Nystatin suspension as positive control showed whole inhibition of the tested *Candida* at MFCs of 440 and 1750 µg/ml for *Candida albicans* MH534933 and *C. glabrata* MH534928, respectively. Conversely, nystatin showed only fungistatic effectiveness against the tested filamentous fungi with MICs (1750 µg/ml).

**Table 3:** Antifungal efficiency of *Spirulina platensis*, sodium selenite (Se), and phyco-synthesized selenium nanoparticles (Se-Nps)

Parameters Tested fungi	MIC µg/ml				MFC µg/ml			
	*S*. *platensis*	Se	Se-NPs	Nystatin	*S*. *platensis*	Se	Se-NPs	Nystatin
*Candida albicans*	9.5	350	350	440	− ve	625	480	440
*C. glabrata*	9.5	625	350	440	− ve	625	580	1750
*Alternaria cerealis*	− ve	625	350	1750	− ve	− ve	950	− ve
*Aspergillus flavipes*	− ve	625	350	1750	− ve	625	580	− ve

− ve: negative effect at the tested concentration

### Anticoagulant activity of Se-NPs

The clot recognition method and the test kits were used to determine the PT and aPTT assay results. The findings of PT and aPTT revealed that freshly prepared Se-NPs more effectively extended the clot formation period when compared to plasma control, sodium selenite, and *S*. *platensis* as presented in Table 4. Where, for PT clot time increased from 12, 19.4, and 14.6 S, respectively to 170.4 S for Se-NPs. For aPTT, clot time increased from 31.2, 60.6, and 42.1 S to 195.6 S for Se-NPs, respectively.

**Table 4:** Anticoagulant activity of Se-NPs against human plasma

Treatments parameters	PT (s)	aPTT (s)
Plasma control	12 ± 0.1	31.2 ± 0.1
Heparin control	350.3 ± 0.15	470.4 ± 0.12
Se-NPs	170.4 ± 0.15	195.6 ± 0.06
Se	19.4 ± 0.1	60.6 ± 0.2
*S*. *platensis*	14.6 ± 0.15	42.1 ± 0.15

Se: sodium selenite, Se-NPs: selenium nanoparticles, *S*. *platensis: Spirulina platensis*, S: seconds

### Cytotoxic potential of SeNPs

**Fig. 9:**
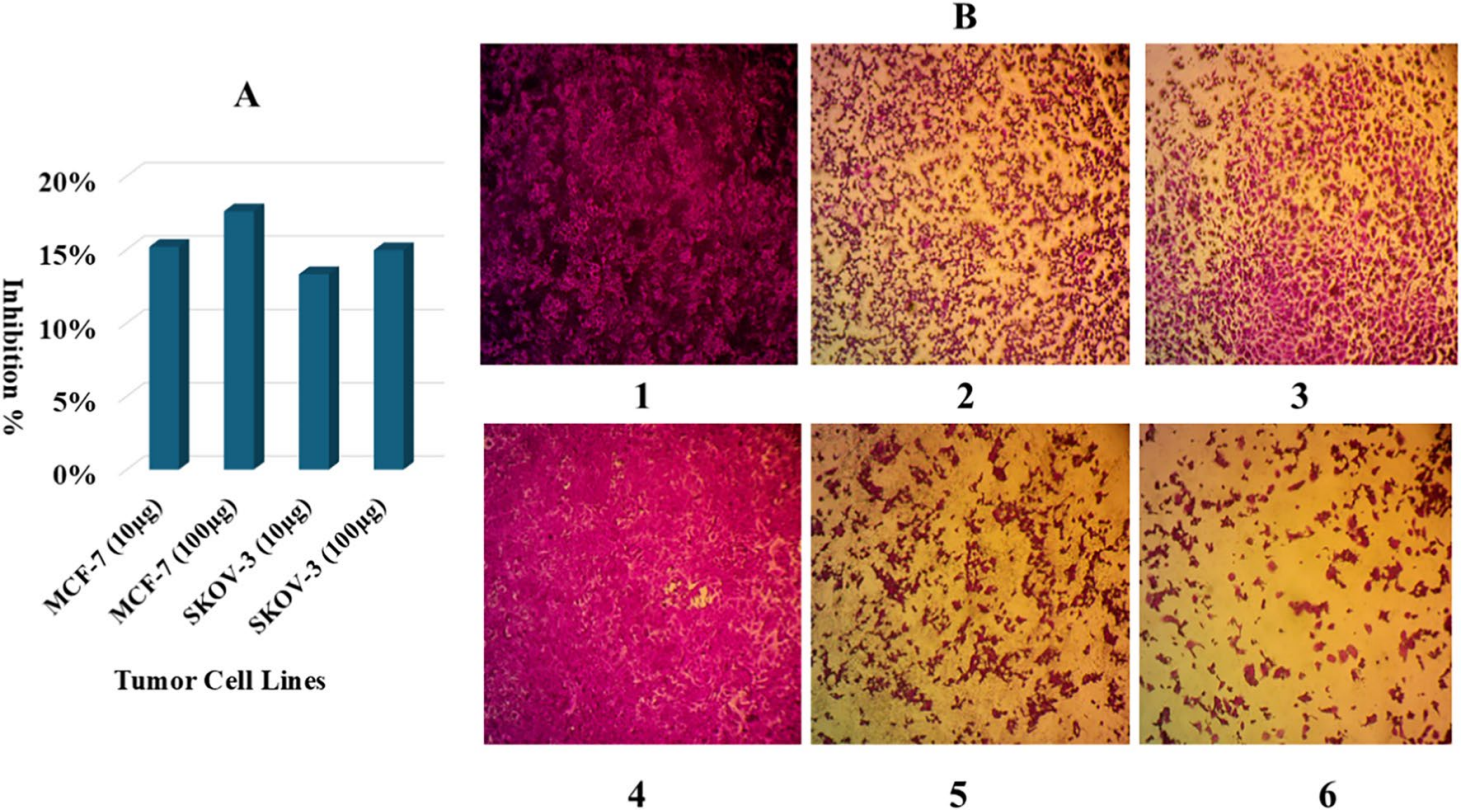
**A:** Inhibition percentage of breast adenocarcinoma (MCF-7) and ovarian tumor (SKOV-3) cell lines at 10 and 100 µg/mL SP-SeNPs. **B:** Cytotoxicity images of SP-SeNPs; 1: MCF-7 control, 2: treated MCF-7 at 10 µg/mL, 3: treated MCF-7 at 100 µg/mL, 4: SKOV-3 control, 5: treated SKOV-3 at 10 µg/mL, 6: treated SKOV-3 at 100 µg/mL

SRB (Quick screening) of SeNPS extract biosynthesized by *S*. *platensis* at concentrations of 10 ug/ml and 100 ug/ml against breast adenocarcinoma (MCF-7) tumor cell line reduced the cell viability to 17.6009% ± 0.918477 at 100 ug/ml and 15.1708% ± 0.949368 at 10 ug/ml (Fig. 9A). The effectiveness of SeNPS extract on ovarian cancer (SKOV-3) cell lines showed a reduction in cell viability of 13.3291% ± 0.568008 and 14.9484% ± 1.028121 at 10 and 100 ug/ml, respectively (Fig. 9A). Treated and untreated MCF-7 and SKOV-3 cell lines micrographs are shown in (Fig. 9B 1, 2, 3, 4, 5, 6).

### Anti-Inflammatory potential of SP-SeNPS

**Fig. 10:**
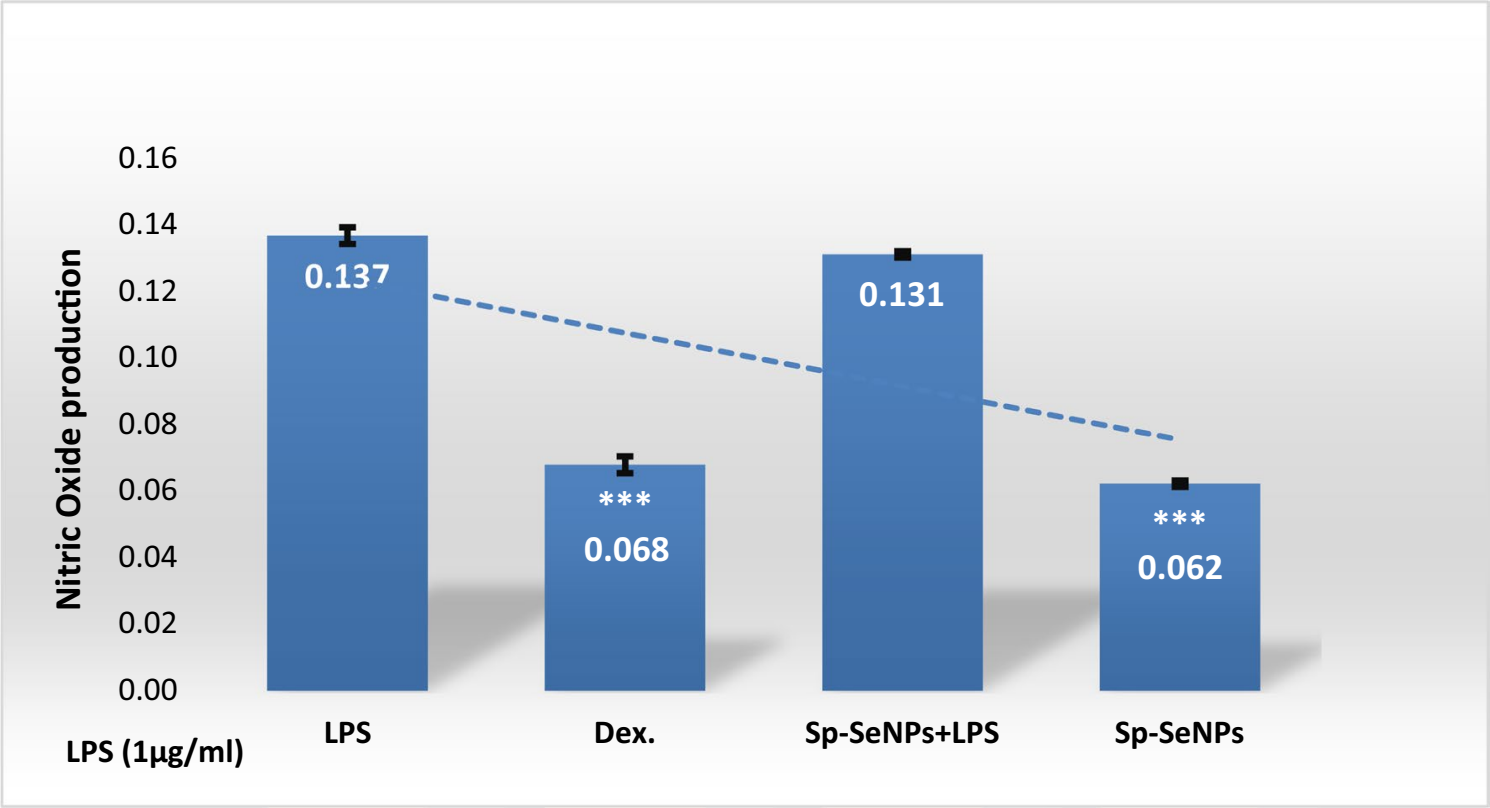
Effects of SP-SeNPS on nitrite production in RAW264.7 cells promoted with 1 µg/mL of LPS. LPS: The control group was treated with 1 µg/mL LPS. Statistical significances were indicated vs. LPS (*** *p* < 0.001)

The nitrite buildup in the culture supernatant indicates that LPS (1 µg/mL) dramatically increased NO generation in macrophages, a marker of an inflammatory response. The NO level was considerably lowered by SP-SeNP treatment in a dose-dependent manner **(**Fig. 10**)**. Numerous pro-inflammatory cytokines and activation substances are generated by immune cells in chronic inflammation to boost the immune system and further involve different inflammatory conditions. The obtained data discovered that SP-SeNPs possess competent anti-inflammatory properties by reducing NO production to 8.82%; this investigation clarified that SP-SeNPs could effectively alleviate the inflammation in RAW 264.7 macrophages.

## Discussion and conclusion

The scientific and pharmaceutical communities have shown a great deal of interest in algae as environmentally friendly, highly nutritious, and supplementary therapeutic sources for secondary metabolites that are bioactive; they have been evaluated as remarkable antioxidants, antimicrobials, and anticancer agents. It's interesting to note that algae metabolites have been suggested as potential options for the environmentally friendly production of biologically active nanoparticles; they function as stabilizing and reducing agents. *Spirulina* extracts have been thoroughly investigated as biologically active substances and bio-synthesizers for potentially medicinal nanoparticles [54]. When combined with *S. platensis* extract, the color of Na_2_SeO_3_ changes from colorless to red color. These findings were consistent with those reported earlier [55], which proposed that the color shift was caused by the SPR being excited. The protein, glucose, fatty acids, and other biomolecules included in the algal extract are believed to aid in converting an inorganic form of sodium selenite (SeIV) to elemental selenium (Se0). Additionally, the capping of the nanoparticles stabilizes the Se nanoparticles with different biomolecules. To create SP-SeNPs, which are insoluble in water, these biomolecules assisted and took part in the reaction with SeO_3_ [56].

Absorption peaks showed the surface plasma resonance of the selenium nanoparticles at the 260 nm region in UV–visible spectroscopy of phyco-synthesized Se-NPs [57]. Following the reduction of selenium ions, the secondary metabolites vanished, and several functional groups were detected by FTIR analysis of the Se-NPs that had been produced. In the FTIR spectrum, The intense and wide peak at 3700–3200 cm^−1^ denotes the N–H, O–H and alcohols stretching vibrations of amide groups respectively, and the bands appearing in the range of 1300–1000 cm^−1^ represent the C-O and C = O stretching vibrations in esters, ethers, or carboxylic acid derivatives, in addition, the peaks at 671.41 cm⁻^1^ corresponds to bending vibrations of C–H in aromatic compounds [58]. Mubarak Ali [59] concurred with this study by confirming the existence of a protein shell that oversees the production of the nanoparticle. The protein that enveloped the selenium nanoparticles served as a stabilizing agent. In a weak reducing environment, hydroxyl and sulfonic groups help create selenium nanoparticles whose particle size is somewhat more significant. The creation of tiny selenium nanoparticles with a narrow particle size distribution is favored by the protein molecule comprising the different functional groups in amino acid sequences, such as the amino, carboxyl, and sulfate groups found in the cyanobacterial protein. Recently, similar to our obtained peaks were detected by FTIR analysis of SeNPs biosynthesized by *Saccharomyces cerevisiae* confirming the presence of amides, amines, and carboxyl groups that indicate the existence of protein molecules surrounding SeNPs [60].

With a mean hydrodynamic size of 411.3 ± 8.529 nm (Z-average) and a polydispersity index (PDI) of 0.548 ± 0.021, the DLS pattern showed a single broad peak, suggesting polydispersity in the nanoparticles' size distribution. SeNPS produced using *Moringa peregrina*, which has an average size of about 400 nm, showed a comparable result [61]. Additionally, the measured zeta potential reached a maximum of -16.7 ± 1.44 mV, indicating stable dispersion in a solution devoid of aggregation; stability is brought about by the large negative charge value on the surfaces of the nanoparticles because of increased particle repulsions [62]. These negatively charged potentials could be called carboxylate polysaccharides and reducing agents (as phenolic compounds) [63]. Because the SEM measures the size of the core nanoparticle, the size distribution varies among SEM measurements and DLS, which measures the molecules that envelop the SeNPs [61].

The TEM identified the biosynthesized SP-SeNPs as agglomerated spheres of smooth surfaces with a diameter of 65 nm. The functional groups from the *S. platensis* aqueous extract adorning the surfaces of the spherical nanoparticles may cause their agglomeration [64]. In line with our results, SeNPs produced with fruit extract from *Zingiber officinale* [65], and leave extract of *Lycium barbarum* [66] were between 100 and 150 nm in size on average.

The obtained XRD patterns show the main peaks characteristic of crystalline-Se that reflects reflections the pure hexagonal phase of selenium crystals. The average crystalline size of Se nanostructures measured by Scherrer’s equation [67] was about 31.74 nm, crystallite size values are in the nano-scale, confirming the nanostructure properties for Se.

In the contemporary investigation, the prepared SeNPs exhibited scavenging of DPPH, ABTS, and H_2_O_2_ radicals, and the scavenging increased as the concentration of the produced Se-NPs samples rose. Apart from the high chemical activity of SeNPs, the small particle size of the nanoparticles makes them dispersible through mediums, which may be the reason for their capacity to neutralize these free radicals [68]. The antioxidant that releases oxygen may enhance the rate at which cells divide, migrate, proliferate, and disperse, promoting healing. Selenium is the major component of glutathione peroxidase antioxidant enzyme scavenging numerous free radicals resulting in liver, heart, and kidney protection from oxidative damage [69]. Several studies confirmed the antioxidant potency of SeNPs by DPPH, ABTS, and H_2_O_2_ assay [60, 70–72]. Therefore, removing free radicals produced by physiological conditions may stop cancer cells from forming and improve healthcare operations [73, 74].

The biosynthesized Se-NPs suppressed the growth of the tested 13 bacterial strains with MBC (286–333 µg/ml). As studied by Guisbiers et al. [75], SeNPs dramatically reduced the number of *S. aureus* and *E. coli* strains after 4, 8, and 24 h without explaining how SeNPs work as antibacterial agents. Zhang et al. [76] demonstrated that Gram-negative bacteria had a significantly higher mortality rate than other bacteria, and SeNPs demonstrated an inhibitory effect against bacterial growth. These results are by the current results where SeNPs inhibited the growth of* Escherichia coli*,* Proteus vulgaris*,* Pseudomonas cepacia*, *Pseudomonas fragi*, *Enterobacter cloace*, *Serratia liquifaciens*, and *Bacillus subtilis*. After interacting with bio-SeNPs, SeNPs changed the membrane's permeability and broke down the cell walls of bacteria, which resulted in the release of polysaccharides and proteins. Additionally, SeNPs' modifications of reactive oxygen species (ROS) intensity may have antimicrobial effects [76]. The antibacterial efficacy of SeNPs on microorganisms has been the subject of conflicting research. The primary cause of this discrepancy between the results of different investigations is the variation in the type of bacteria used and the size of the nanoparticles. Since smaller nanoparticles produced more ROS inside or outside of cells than larger ones with a surface area to volume ratio [77].

The manufactured selenium nanoparticles markedly suppressed all examined bacterial strains' ability to produce biofilms. Numerous investigations have demonstrated how nanoparticles work as biofilm inhibitors against various microorganisms. These outcomes agree with a prior study conducted by Shakibaie et al. [78], which found that SeNPs therapy reduced *P. mirabilis*, *S. aureus*, and *P. aeruginosa* biofilm development by 53.4, 42, and 34.3%, respectively.

The biosynthesized selenium nanoparticles exposed a promising antifungal potency compared with *S*. *platensis*, sodium selenite extracts, and nystatin as a positive control against the tested *Candida* and molds. Our presented results following a recent investigation by Eydelkhani et al. [79] found that SeNPs biosynthesized by *Alborzia kermanshahica* cyanobacteria cultured in control conditions exhibited low antifungal efficacy than those under 60 m T magnetic field and *Aspergillus niger* was more susceptible than *Penicillium chrysogenum*. Abdel-Moneim et al. [80] mentioned that SeNPs and *Spirulina* exhibited antifungal efficacy against the tested molds and yeast. *Cryptococcus neoformans, Candida tropicalis*,* C. albicans*,* C. krusei*,* C. parapsilosis*, and *C. glabrata* were suppressed by green synthesized SeNPs [81, 82]. Using the disk diffusion method, SeNPs inhibited the growth of *Fusarium mangifera* at varying concentrations. The 300 µg/ml concentration produced the largest inhibition zone diameter, 14 mm. [83]. *Alternaria alternata*, *Botrytis cinerea*, and *Phytophthora capsid* were the most sensitive to SeNPs biosynthesized by *Desmonostoc alborizicum*. On the other hand, *Rhizopus stolonifer*, *Fusarium oxysporum*, *Colletotrichum gloeosporioides*, and *Pythium ultimum* were resistant. [84]. The antifungal efficiency of biogenic SeNPs may return to cell membrane lysis, inhibition of amino acid development, and prevention of DNA replication due to the over-expression of ROS [85].

Anticoagulant findings of PT revealed that freshly prepared Se-NPs extended the time taken for the clot to form more effectively than plasma control, sodium selenite, and *S*. *platensis*. PT and PTT blood clotting normal times were from 11 to 15 S and 25 to 53 S, respectively [86, 87]. Blood in the vasculature clots quickly and remains fluid when it encounters subendothelial surfaces. Under normal circumstances, coagulation/fibrinolysis equilibrium avoids bleeding and thrombosis. Any disparity promotes coagulation, which results in fibrin formation, platelet aggregation, thrombosis, and red blood cells stuck in veins or arteries. To treat thrombosis, several antithrombotic drugs are available. Anticoagulant drugs stop the creation of fibrin, while antiplatelet drugs stop platelets from activating or aggregating. However, fibrinolytic therapies break down the formation of fibrin. [88]. Recent research has demonstrated that employing biologically generated Se-NPs rather than selective active molecules results in efficient anticoagulant effects while utilizing fewer active molecules [49, 89]. SeNPs synthesized with *S. platensis* in this study improved anticoagulant properties, but not as much as heparin, the common anticoagulant medication Table 4. This could be because they can prevent prothrombin from being converted to thrombin, which is a crucial phase in which various clotting agents are catalyzed and insoluble fibrin strands are formed, as stated by Jin and Gopinath [90].

The antitumor properties of the tested SeNPs extract against SKOV-3 and MCF-7 cell lines were evaluated by SRB (Quick screening). SeNPs have shown a low cytotoxic effect on normal cells compared with Se^+4^ and Se^+6^ ions and their cytotoxic influence is related to several mechanisms such as DNA destruction, formation of ROS, disturbance of cellular homeostasis, apoptosis, and death of cancer cells [91, 92]. Green SeNPs by *Bacillus coagulant* showed cytotoxic activity with IC50 of 17.56 µg/ml against cancer cells of the breast [93]. SeNPs decorated by *Spirulina* polysaccharides showed significant cytotoxicity against MCF-7, Hela-229, MG-63, and A375 tumor cell lines [94]. SeNPs inhibited SKOV-3 ovarian cell monolayer growth, but the inhibition was higher in the case of selenite with IC20 (3 µg/ml) compared with 6 and 13 µg/ml for seNP-BSA and SeNP-chitosan, respectively [95].

This investigation illustrated that SP-SeNPs could effectively alleviate the inflammation in RAW 264.7 macrophages, as demonstrated by Ansari et al. [96], who illustrated that It has been demonstrated that selenium NPs have biological effects, including reducing inflammation by hindering mRNA synthesis.

In conclusion, these findings could offer scientific evidence for the application and advancement of these algal cell-synthesized nanoparticles as a complementary therapy for the avoidance of inflammation and disorders associated with it, including cytotoxicity, fungistatic, bacteriostatic, anti-biofilm, and anticoagulant effects.

## Data Availability

All data obtained during this investigation are incorporated in this manuscript.
